# Custodiol versus blood cardioplegia in pediatric cardiac surgery: a randomized controlled trial

**DOI:** 10.1186/s40001-023-01372-4

**Published:** 2023-10-05

**Authors:** Ahmed F. Elmahrouk, Mohammad S. Shihata, Osman O. AL-Radi, Amr A. Arafat, Musleh Altowaity, Bayan A. Alshaikh, Mohamed N. Galal, Abdulbadee A. Bogis, Haneen Y. Al Omar, Wesal J. Assiri, Ahmed A. Jamjoom

**Affiliations:** 1 Cardiothoracic Surgery Department, King Faisal Specialist Hospital and Research Centre,, MBC J-16, P.O. Box: 40047, 21499 Jeddah, Saudi Arabia; 2 Cardiothoracic Surgery Department, Tanta University, Tanta, Egypt; 3 Department of Surgery, Cardiac Surgery Section, King Abdulaziz University, Jeddah, Saudi Arabia; 4 Cardiac Surgery Department, King Fahad Armed Forces Hospital, Jeddah, Saudi Arabia; 5 Pediatric Cardiac Surgery Department, King Fahad Armed Forces Hospital, Jeddah, Saudi Arabia; 6 Research Centre, King Faisal Specialist Hospital and Research Centre, Jeddah, Saudi Arabia; 7 Department of Nursing, King Faisal Specialist Hospital and Research Centre, Jeddah, Saudi Arabia

**Keywords:** Cardioplegia, Custadiol, Blood cardioplegia, Histidine-tryptophan-ketoglutarate

## Abstract

**Background:**

Blood-based cardioplegia is the standard myocardial protection strategy in pediatric cardiac surgery. Custadiol (histidine-tryptophan-ketoglutarate), an alternative, may have some advantages but is potentially less effective at myocardial protection. This study aimed to test whether custadiol is not inferior to blood-based cardioplegia in pediatric cardiac surgery.

**Methods:**

The study was designed as a randomized controlled trial with a blinded outcome assessment. All pediatric patients undergoing cardiac surgery with cardiopulmonary bypass and cardioplegia, including neonates, were eligible. Emergency surgery was excluded. The primary outcome was a composite of death within 30 days, an ICU stay longer than 5 days, or arrhythmia requiring intervention. Secondary endpoints included total hospital stay, inotropic score, cardiac troponin levels, ventricular function, and extended survival postdischarge. The sample size was determined a priori for a noninferiority design with an expected primary outcome of 40% and a clinical significance difference of 20%.

**Results:**

Between January 2018 and January 2021, 226 patients, divided into the Custodiol cardioplegia (CC) group (*n* = 107) and the blood cardioplegia (BC) group (*n* = 119), completed the study protocol. There was no difference in the composite endpoint between the CC and BC groups, 65 (60.75%) vs. 71 (59.66%), respectively (*P* = 0.87). The total length of stay in the hospital was 14 (*Q*2–*Q*3: 10–19) days in the CC group vs. 13 (10–21) days in the BC group (*P* = 0.85). The inotropic score was not significantly different between the CC and BC groups, 5 (2.6–7.45) vs. 5 (2.6–7.5), respectively (*P* = 0.82). The cardiac troponin level and ventricular function did not differ significantly between the two groups (*P* = 0.34 and *P* = 0.85, respectively). The median duration of follow-up was 32.75 (*Q*2–*Q*3: 18.73–41.53) months, and there was no difference in survival between the two groups (log-rank *P* = 0.55).

**Conclusions:**

Custodial cardioplegia is not inferior to blood cardioplegia for myocardial protection in pediatric patients.

*Trial registration* The trial was registered in Clinicaltrials.gov, and the ClinicalTrials.gov Identifier number is NCT03082716 Date: 17/03/2017

**Supplementary Information:**

The online version contains supplementary material available at 10.1186/s40001-023-01372-4.

## Introduction

Cardioplegia is an essential component of cardiac surgery that allows surgery on the arrested heart with preservation of the myocardium [1–3]. Despite years of investigations, the optimal cardioplegic solution for pediatric cardiac surgery has not been reached, and there is wide variability in practice worldwide [4, 5]. Blood-based cardioplegia has proven efficacy and is preferred for myocardial protection in the pediatric population [6, 7]. However, blood cardioplegia has the drawbacks of repeated dosing, resulting in prolonged operative time and interrupted surgical repair [7].

Custodiol (histidine–tryptophan–ketoglutarate (HTK) solution) Dr. Franz Köhler Chemie GmbH) was first introduced in 1970 for myocardial protection and organ preservation. Custodiol is an intracellular, crystalloid cardioplegia solution that is given in a single dose that lasts for 3 h [8]. The use of Custodiol in pediatric cardiac surgery is not popular, and there is a paucity of studies comparing Custodiol vs. blood cardioplegia in pediatric patients [9]. Currently, the efficacy and safety of Custodiol over blood cardioplegia in pediatric cardiac surgery have not been proven. Therefore, the objective of this study was to assess whether Custadiol is not inferior to blood cardioplegia in pediatric cardiac surgery.

## Methods

### Design and patients

**Fig. 1:**
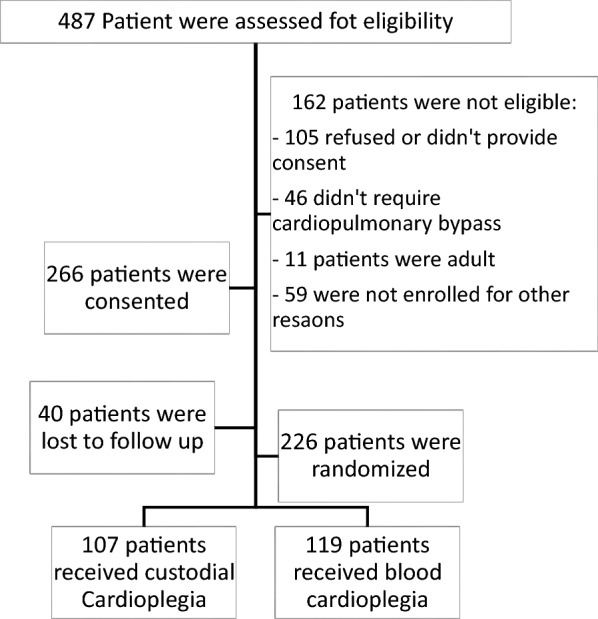
Study flowchart

We conducted a randomized clinical trial to compare the outcomes of Custodiol cardioplegia (CC) vs. blood cardioplegia (BC) in pediatric cardiac surgery. The study was conducted between January 2018 and January 2021 in King Faisal Specialist Hospital and Research Centre-Jeddah, Saudi Arabia. The data were collected using approved CRFs, and all the data were transferred to REDCap software for management. However, the trial stopped due to COVID-19 and the difficulty of enrollment at the time, and only 487 patients were assessed for eligibility. We included pediatric patients who had a repair on cardiopulmonary bypass and cardioplegia, and neonates were eligible. We excluded adult patients with congenital heart disease, patients who refused to consent (or their guardians), emergency cases, and patients who were lost to follow-up. The study flowchart is presented in Fig. 1. The patients were divided into the Custodiol cardioplegia group (*n* = 107) and the blood cardioplegia group (*n* = 119).

### Ethical considerations

The study was approved prior to patient recruitment by the Institutional Review Board of King Faisal Specialist Hospital and Research Center, Jeddah (Approval# 2016–8). All study activities and methods were in accordance with ICH-GCP and local regulatory guidelines. Informed consent for retention and use of patient data for research purposes was obtained from their guardians at the time of the procedure consent before randomization. The patients or their guardians retained the right to withdraw from the study at any time.

### Safety monitoring and interim analysis

Since both cardioplegia solutions are already in use and FDA-approved, there are no safety concerns at the time being. However, an interim analysis will be carried out every 6 months, and the principal investigator will decide whether to stop or carry on if any major discrepancies in outcomes are observed.

### Randomization

The day before the surgery, the study team approached eligible patients for enrollment. They were randomly assigned to one of two study arms using computer-generated simple random samples.

### Intervention

#### Blood cardioplegia group

After cross-clamping, the patient received blood cardioplegia, delivered using the microplegia delivery system by adding potassium to the blood (*K* = 35 ml eq/L). The initial dose was 35 ml/kg, and subsequent doses of 20–15 ml/kg were given every 20 min at a temperature of 10–15 °C while maintaining a perfusion pressure of 100–125 mmHg.

#### Custodiol group

After the cross-clamping patient received a single dose of HTK Custodiol cardioplegia at a temperature of 4–8 °C and was perfused for 6–8 min, the dose was started from 400 to 1000 ml according to the child's body weight (35 ml/kg). Perfusion pressure was kept at 70–80 mmHg until the heart was arrested Additional file 1.

### Blinding

Triple blinding (participants, investigators, outcome assessors) was used. Surgeons knew the type of cardioplegia in the operating room, while other assessors were blinded. The research coordinator generated the random allocation sequence, and the investigators enrolled the patients while study surgeons assigned the interventions to the patients.

### Study outcomes

The primary outcome was a composite of 30-day mortality, ICU stay of more than 5 days, and postoperative arrhythmia occurring within 48 h of surgery and requiring intervention.

Secondary endpoints were the length of stay (days), length of mechanical ventilation (days), myocardial biomarkers (troponin) measured (preoperatively, 6 h, 24 h, 48 h postoperatively), ventricular function assessed by fractional shortening and ejection fraction, extracorporeal membrane oxygenation support (ECMO) and vasoactive inotropic score [dopamine dose (μg/kg/min) + dobutamine dose (μg/kg/min) + 100*X* epinephrine dose (μg/kg/min) + 10*X* milrinone dose (μg/kg/min) + 100*X* norepinephrine dose (μg/kg/min) + 10,000*X* vasopressin dose (U/kg/min)].

### Sample size

The sample size was determined a priori for a noninferiority design with an expected primary outcome of 40% and a clinical significance difference of 20%.

### Statistical analysis

Categorical data are presented as numbers and percentages and were compared with the chi-squared test or Fisher’s exact test if the expected frequency was less than five. Continuous data were expressed as the median and (*Q*1–*Q*3) and compared with the Mann‒Whitney test. The random effect model was used to compare the changes in troponin levels between groups. A Kaplan‒Meier curve was used for survival distribution, which was compared with the log-rank test. Stata 16.1 was used for analysis (Stata Corp- College Station- TX- USA).

## Results

### Preoperative and operative data

There were no differences in age, gender, body surface area, preoperative left and right ventricular function, ventricular repair, cyanotic heart disease, or preoperative troponin levels between the groups (Table 1).

**Table 1:** Comparison of preoperative data between patients who received Custodiol vs. blood cardioplegia

	Custodiol Cardioplegia (*n* = 107)	Blood cardioplegia (*n* = 119)	*P*-value
Male, *n* (%)	51 (47.66)	65 (55.08)	0.27
Age (m), median (*Q*1–*Q*3)	8 (5–36)	10 (5–21)	0.48
Weight (kg), median (*Q*1–*Q*3)	6.5 (5.1–11.6)	6.5 (4.9–9.8)	0.80
BAS (m^2^), median (*Q*1–*Q*3)	0.34 (0.28–0.60)	0.35 (0.28–0.47)	0.79
Preoperative LV function, *n* (%)^a^	(*n* = 101)	(*n* = 116)	0.51
Normal	97 (96.04)	110 (94.83)	
Mildly depressed	2 (1.98)	5 (4.31)	
Moderately depressed	2 (1.98)	1 (0.86)	
Preoperative RV function, *n* (%)^a^	(*n* = 101)	(*n* = 115)	0.85
Normal (EF: 55–69%)	92 (91.09)	107 (93.04)	
Mildly depressed (EF: 45–50%)	4 (3.96)	5 (4.35)	
Moderately depressed (EF: 35–39%)	4 (3.96)	3 (2.61)	
Moderately to severely depressed (EF: 30–34%)	1 (0.99)	0	
Ventricular repair, *n* (%)			0.31
Biventricular	100 (93.46)	114 (95.80%)	
Uni-ventricular	7 (6.54)	5 (4.20%)	
Cyanotic heart disease	24 (22.43%)	32 (26.89%)	0.43
Preoperative troponin (*U*), median (*Q*1–*Q*3)	9.6 (4.6–16.5)	11 (5.1–26.8)	0.13

EF: ejection fraction; LV: left ventricle, RV: right ventricle

^a^Ventricular function was measured with transthoracic echocardiography

The ischemic and cardiopulmonary bypass times, lowest core temperature, and cardioplegia temperature did not differ significantly between groups (Table 2).

**Table 2:** Comparison of operative data between patients who received Custodiol vs. blood cardioplegia

	Custodiol Cardioplegia (*n* = 107)	Blood cardioplegia (*n* = 119)	*P* value
CPB time (min), median (*Q*1–*Q*3	77 (52–106)	76 (49–107)	0.64
Ischemic time (min), median (*Q*1–*Q*3)	46 (32–79)	51 (27–69)	0.66
Lowest core temperature (C), median (*Q*1–*Q*3)	32 (32–33)	32 (32–33)	0.96
Cardioplegia temperature (C), median (*Q*1–*Q*3)	4 (4–6)	4 (4–6)	0.51

CPB: cardiopulmonary bypass time

### Postoperative data

There was no difference in the composite endpoint between the CC and BC groups, 65 (60.75%) vs. 71 (59.66%), respectively, *P* = 0.87. The total length of stay in the hospital was 14 (25–75th percentiles: 10–19) days in the CC group vs. 13 [10] days in the BC group, *P* = 0.85. The vasoactive inotropic score was not significantly different between the CC and BC groups; VIS was 5 (2.6–7.45) vs. 5 (2.6–7.5) in the CC vs. BC groups, respectively (*P* = 0.82). There was no difference in postoperative arrhythmia between groups (*P* = 0.87). The most common types of arrhythmia were junctional ectopic tachycardia (*n* = 23, 41.07%) and complete heart block (*n* = 22, 39.29%) (Table 3).

**Table 3:** Comparison of postoperative data between patients who received Custodiol vs. blood cardioplegia

	Custodiol Cardioplegia (*n* = 107)	Blood cardioplegia (*n* = 119)	*P* value
Delayed sternal closure, *n* (%)	17 (15.89)	19 (15.97)	0.99
Arrythmia^a^	26 (24.30)	30 (25.21)	0.87
Postoperative LV function, *n* (%)	(*n* = 99)	(*n* = 113)	0.85
Normal	63 (63.64)	71 (62.83)	
Mildly depressed	21 (21.21)	28 (24.78)	
Moderately depressed	12 (12.12)	11 (9.73)	
Moderately to severely depressed	2 (2.02)	3 (2.65)	
Severely depressed	1 (1.01)	0	
Vasoactive inotropic score, median (*Q*1–*Q*3)^b^	5 (2.6–7.45)	5 (2.6–7.5)	0.82
Duration of MV (*h*), median (*Q*1–*Q*3)	19.45 (5–48)	21 (5–55.4)	0.50
ECMO, *n* (%)	3 (2.8)	2 (1.68)	0.67
ICU stay (*d*), median (*Q*1–*Q*3)	6 (3–9)	6 (4–11)	0.33
Hospital stay (*d*), median (*Q*1–*Q*3)	14 (10–19)	13 (10–21)	0.85
30-day mortality	1 (0.93)	3 (2.52)	0.62

ECMO: extracorporeal membrane oxygenation; ICU: intensive care unit; LV: left ventricle; MV: mechanical ventilation

^a^Defined as arrythmia occurring within 48 h and requiring intervention

^b^Vasoactive inotropic score calculated at 48 h

**Fig. 2:**
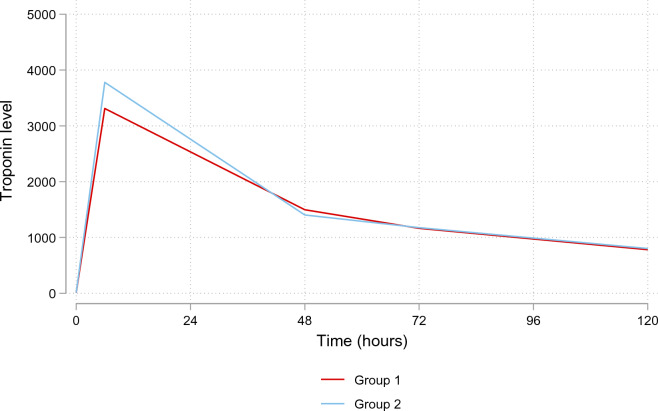
Change in troponin levels in patients with Custodiol (Group 1) vs. blood cardioplegia (Group 2)

**Fig. 3:**
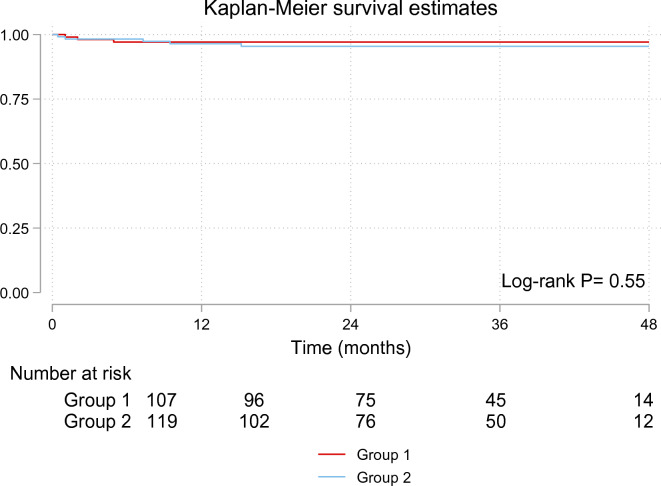
Kaplan–Meier survival curve of patients with Custodiol (Group 1) vs. blood cardioplegia (Group 2)

The cardiac troponin level and ventricular function did not differ significantly between the two groups, *P* = 0.34 and *P* = 0.85, respectively (Fig. 2). The median duration of follow-up was 32.75 (Q2–Q3: 18.73–41.53) months. Mortality occurred in 3 patients in the CC group and five patients in the BC group, and there was no difference in survival between the two groups (log-rank *P* = 0.55) (Fig. 3).

## Discussion

The debate about the optimal cardioplegic solution in pediatric cardiac surgery continues [10]. Studies comparing different cardioplegic solutions in pediatric cardiac surgery have used different endpoints on a small number of patients [10]; therefore, the results are variables, and conclusions about the optimal cardioplegic solution cannot be reached. Global practice varies widely, and new cardioplegic solutions have been introduced with variable outcomes [8, 11–13].

In this clinical trial, randomized patients to receive either Custodiol cardioplegia (*n* = 107) or blood cardioplegia (*n* = 119). The primary outcome was a composite of death within 30 days, an intensive care stay longer than five days, or arrhythmia requiring intervention. We did not report differences between both groups in the primary or secondary outcomes. The type of cardioplegia did not affect the level of troponin or survival in our sample.

Blood cardioplegia is the standard of care in pediatric cardiac surgery [14]; however, there is a tendency to shift to single-dose cardioplegia to minimize the interruption of surgical operation [15]. Therefore, intracellular solutions such as Custodiol and Del-Nido cardioplegia are currently increasingly used in pediatric cardiac surgery [16]. Bibevski and associates found no difference between Custodiol and blood cardioplegia in left and right ventricular function; however, the inotropic support was lower with Custodiol cardioplegia [9]. On the other hand, Liu and associates found no difference in inotropic support between Custodiol and St. Thoms cardioplegia [17].

The reported effect of Custodiol cardioplegia on mortality in the literature is controversial. Lin and colleagues reported lower mortality rates in patients who received Custodiol cardioplegia compared to St. Thomas [18]. Giordano and colleagues [19] and Bojan and coworkers [20] found no difference in the clinical outcomes between Custodiol and blood cardioplegia. In a meta-analysis of 12 studies containing 1,634 pediatric patients, four types of cardioplegia were compared [16]. The study reported no difference in mortality between Custodiol, Del-Nido, and St. Thomas and blood cardioplegia.

Our study showed that Custodiol cardioplegia had a similar risk profile compared to blood cardioplegia in pediatric cardiac surgery. However, continuous research is recommended to reach the optimum cardioplegic solution in children.

### Study limitations

We included different age groups and patients who had one-ventricle and two-ventricle repair. This wide variability of the preoperative characteristics could have affected the outcomes. We used simple randomization that could have created an imbalance in the number of patients between groups. Last, our center is a tertiary referral center; not all patients were available for follow-up. Some patients were foreigners and were unavailable in the country after surgical repair.

### Strength

On the other hand, this study evaluated the effect of cardioplegia on different clinical, laboratory, and echocardiographic outcomes, which is not frequently reported in the literature. Additionally, the study reported long-term mortality in those patients with a median follow-up of 33 months.

## Conclusion

Custodial is not inferior to blood cardioplegia for myocardial protection in pediatric patients; especially in patients with biventricular repair with short aortic clamping time.

### Supplementary Information

**Additional file 1.** The study protocol, including overview of the study and detailed discrption.

## Data Availability

Unidentified data are available upon request with the corresponding author.
