# Supplementary material for: Custodiol versus blood cardioplegia in pediatric cardiac surgery: a randomized controlled trial
Source: Eur J Med Res. 2023 Oct 5;28:404. doi: 10.1186/s40001-023-01372-4 (PMC10552411; doi:10.1186/s40001-023-01372-4)
Supplement: Supplementary file 1 — Additional file 1. The study protocol, including overview of the study and detailed discrption. [file 40001_2023_1372_MOESM1_ESM.docx]

**Custodiol Versus Blood Cardioplegia in Pediatric Cardiac Surgery**

**Research Proposal IRB # 2016-08**

**Study Overview**

**Brief Summary:**

Cardioplegic arrest is an essential part of cardiac surgery which aims to allow myocardial preservation and minimize myocardial swelling, while providing a motionless and bloodless field, Blood cardioplegia has proven its efficacy for several decades and surgeons are still preferring to use it for myocardial protection of pediatric cardiac surgery, although it is thought to be more time consuming since it is given with interrupted doses, Even when advancement has come along the field of myocardial protection and cardioplegia solutions with the introduction of Bretschneider Histidine-Tryptophan-Ketoglutarate solution , custodiol ,in 1970 ,which is given as single dose and believed to be convenient, simple to deliver , and less time consuming .Many Surgeons haven't change their practice possibly due to paucity of studies comparing cardioplegia solutions in pediatric cardiac surgery and conflicting reports regarding the superiority of different cardioplegia solution. The investigators aim to provide evidence that will help pediatric cardiac surgeons to choose the optimal solution for their practice.

**literature review:** After an extensive literature review the investigators found only one randomized trial by Elmorsy et al, which concluded that A single dose of an HTK cardioplegic solution provides better myocardial and cerebral protection than repeated doses of oxygenated blood cardioplegia during pediatric congenital cardiac surgery.

On the other hand, there was another retrospective report by bojan et al, found that the use of Custodiol cardioplegia in neonates undergoing ASO was associated with a larger troponin release when compared with warm blood cardioplegia, p <0.001 suggesting poor myocardial protection.

Koruon et al, done a tissue level comparison between htk and conventional crystalloid cardioplegia and their statistical analysis revealed no significant difference between the two groups regarding the clinical variables, apoptotic indices and proliferation indices.

Liu j et al, did a retrospective study comparing custodiol to St. Thomas crystalloid cardioplegia which concluded That HTK group had shorter cross-clamping time and more frequent spontaneous defibrillation than St. Thomas group.

Also, an animal study by Chen Y et al, on piglets that were randomized to either a single dose of HTK or multidose cold blood cardioplegia. No significant differences were noted in the myocardial lactate content, ATP content, and histopathological score between both groups.

Finally, a retrospective report by E Qulisy et al concluded that Custodiol cardioplegia is associated with less myocardial protection and higher adverse outcomes compared to blood cardioplegia in pediatric age group undergoing cardiac surgery.

**Detailed Description:**

**Hypothesis**:

Custodial is not inferior to blood cardioplegia in myocardial protection in pediatric cardiac surgery.

**Methodology:**

**Study design:**

Randomized controlled trial

**Data collection**:

All demographic data, diagnosis, procedure (type. CPB time, cross-clamp time), surgeon and hospital course details.

**Inclusion criteria:**

- All pediatric patients (10 to 18y) referred to our hospital which will undergo open cardiac repair with cardiopulmonary bypass

**Exclusion criteria:**

- All emergency procedure (require immediate surgery)
- Cases that don't require use of cardioplegia.

**Randomization:**

Computer generated randomization into two groups based on cardioplegia solution. if randomized to blood patient will receive blood cardioplegia, delivered by microplegia delivery system by adding potassium to the blood (K= 35 ml eq/L). The initial dose will be 35ml/ kg, and subsequent doses 20-15 ml/kg given every 20 minutes at a Temperature of 10 - 15 C, while maintaining a perfusion pressure of 100-125 mmg. if randomized to custodial patient will receive single dose of HT custodiol cardioplegia. at temperature of 4-8°C and will be perfused for 6-8 minutes. Dose will start from 400 up to 1000 ml according to the child's body weight. Perfusion pressure will be kept at 70 - 80 mmHg until the heart is arrested.

**Blinding:**

Surgeons will know the type of cardioplegia in the OR while another assessor will be blinded.

Sample size calculated with an online calculator based on α _error of 0.05 and β _error of0.2 to detect an absolute risk difference of 15% in composite end point between the twogroups yielded 137 in each arm, the results will be presented as mean (SD), median

(interquartile range), or proportion. Using relative risk and log rank for statistical analysis. A P value of less than 0.05 will be considered statistically significant. Statistical analyses will be carried out using the SPSS.

**Outcomes:**

Primary end point will be composite of mortality (30d); ICU stay more than 5daysand post-operative arrhythmia requiring intervention (48h). Secondary end point length of hospital stays(days), length of mechanical ventilation (days), myocardial biomarkers (troponin, CKMB), ventricular function by echo, CMO, inotropic score sample size calculated with an online calculator basis of an error of 0.05 and a B error of 0.2 to detect an absolute difference of 15% in composite end point between the two gps yielded 137 in each arm, the results will be presented as mean (SD), median (interquartile (range), or proportion. Using relative risk for statistical analysis and log rank. A P value of less than 0.05 will be considered statistically significant. Statistical analyses will be carried out using the SPSS

**Safety monitoring and interim results:**

Both cardioplegia solutions are already in use and FDA approved there is no safety concerns at the time being. However, an interim analysis will be carried on every 6months and the principal investigator will decide whether to stop or carry on if any major discrepancies in outcomes are observed

**References (comprehensive literature review)**

1. ﻿﻿﻿﻿Kotani, Yasuhiro, et al. "Current cardioplegia practice in pediatric cardiac surgery: a North American multinstitutional survey." The Annals of thoracic surgery 96.3 (2013): 923-929.
2. ﻿﻿﻿Harvey B, Shann KG, Fitzgerald D, et al. International Pediatric Perfusion Practice: 2011 Survey Results. The Journal of Extra-corporal Technology. 2012;44(4):186-193.
3. ﻿﻿﻿﻿elmorsy et al, Does type of cardioplegia affect myocardial and cerebral outcome in pediatric open cardiac surgeries? Ain-Shams Journal of Anesthesiology ,2014; 2(7) :242-249
4. ﻿﻿﻿﻿Bojan, Mirela, et al. "Cold histidine-tryptophan-ketoglutarate solution and repeated oxygenated warm blood cardioplegia in neonates with arterial switch operation." The Annals of thoracic surgery 95.4 (2013):1390-1396.
5. ﻿﻿﻿kouron et al , The comparison of the effects of Bretschneider's histidine-tryptophan-ketoglutarate and conventional crystalloid cardioplegia on pediatric myocardium at tissue level. ASAIO J. 2008 Sep-Oct;54(5):470-3
6. ﻿﻿﻿﻿liu jet al, The myocardial protection of HTK cardioplegic solution on the long-term ischemic period in pediatric heart surgery."ASAIO J. 2013 Jan-Feb: 59(1):69-74
7. ﻿﻿﻿Chen Yet al, Which is the better option during neonatal cardiopulmonary bypass: HTK solution or cold blood cardioplegiaASAIO J. 2013 Jan-Feb:59(1):69-74
8. ﻿﻿﻿﻿Custodio versus blood cardioplegia in pediatric cardiac surgery. SHAJ .October 2015;27, (4):327
